# Characterization and control of *Rhizoctonia solani* affecting lucky bamboo (*Dracaena sanderiana* hort. ex. Mast.) using some bioagents

**DOI:** 10.1038/s41598-023-33628-8

**Published:** 2023-04-24

**Authors:** Taghreed F. M. Abdel-Rahman, Ahmed Abdel-Megeed, Mohamed Z. M. Salem

**Affiliations:** 1 Department of Ornamental, Medicinal and Aromatic Plant Diseases, Plant Pathology Research Institute, Agricultural Research Center (ARC), Giza, 12619 Egypt; 2 Department of Plant Protection, Faculty of Agriculture (Saba Basha), Alexandria University, Alexandria, 21531 Egypt; 3 Forestry and Wood Technology Department, Faculty of Agriculture (El-Shatby), Alexandria University, Alexandria, 21545 Egypt

**Keywords:** Biological techniques, Microbiology, Plant sciences

## Abstract

In a survey conducted during the period of March–May 2019 in nurseries, warehouses, and shops at three governorates (Alexandria, El-Behera, and Giza governorates, Egypt), symptoms of root rot, basal stem rot, and wilt disease complex were observed in the lucky bamboo (*Dracaena sanderiana* hort. ex. Mast.). The highest disease infection percentage was found in lucky bamboo collected from Alexandria City (47.67%), while the highest disease severity was in lucky bamboo collected from El-Behera Governorate (35.19%). *Rhizoctonia solani*,* Fusarium oxysporum*, *F. solani*, *Aspergillus niger*, and *Alternaria alternate* were isolated and identified in the infected lucky bamboo samples. *R. solani* isolates were the most dominant among the recovered fungal species with a percentage of 80.89% of the total isolates (246). Pathogenicity tests showed that *R. solani* was the most pathogen with 100% disease infection and 76.67% disease severity. Molecular identification characterized *R. solani* isolate as *R. solani* AUMC 15120, MZ723906. Meanwhile, four biological control agents (bioagents) were isolated from the healthy lucky bamboo samples and identified based on cultural, morphological, microscopic characteristics, and the molecular phylogenetic analysis as *Clonostachys rosea* AUMC 15121, OL461708; *Bacillus circulans* TAG1, MW441316; *B. siamensis* TAP1, MW441318 and *Ochrobactrum anthropi* TAM1, MW441317. The four bioagents showed potential inhibition of *R. solani *in vitro as well as in vivo on lucky bamboo plants in vase treatments compared to the untreated inoculated control as well as certain fungicides and biocides used (Moncut, Rizolex-T, Topsin-M, Bio-Zeid, and Bio-Arc)*.* The bioagent *O. anthropi* showed the highest inhibition growth (85.11%) of the in vitro* R. solani* colony, which was not significantly different from the biocide Bio-Arc (83.78%). However, *C. rosea*, *B. siamensis* and *B. circulans* showed inhibition values of 65.33, 64.44, and 60.44%, respectively. On the other hand, the biocide Bio-Zeid showed less inhibitory effect (43.11%), while the lowest growth inhibition was recorded by Rizolex-T (34.22%) and Topsin-M (28.67%). Furthermore, the in vivo experiment supported the in vitro results for the most effective treatments, where all the treatments significantly decreased the percentage of infection and disease severity compared to the inoculated untreated control. Additionally, the bioagent *O. anthropi* showed the highest effect, *i.e*., the lowest disease incidence and disease severity being 13.33% and 10%, compared to 100% and 75%, respectively, in the untreated inoculated control. This was not significantly different from the fungicide Moncut (13.33% and 21%) and from the bioagent *C. rosea* (20% and 15%) treatments for both parameters, respectively. In conclusion, the bioagents *O. anthropi* MW441317 at 1 × 10^8^ CFU/ml as well as *C. rosea* AUMC15121 at 1 × 10^7^/ml proved to be efficient to control *R. solani* causing root rot, and basal stem rot on lucky bamboo, compared to fungicide Moncut and can be used for disease management without the negative impact of the chemical control. Furthermore, this is the first report of the isolation and identification of *Rhizoctonia solani*, a pathogenic fungus, and four biocontrol agents (*Bacillus circulans*, *B. siamensis*, *Ochrobactrum anthropi* and *Clonostachys rosea*) associated with the healthy lucky bamboo plants.

## Introduction

*Dracaena sanderiana* hort. ex. Mast., belongs to the Asparagaceae family, and is commonly known as "lucky bamboo"^1^. It is not bamboo (Poaceae family), but the stems resemble real bamboo stalks. *D. sanderiana* is one of the most vase plants and is widely used in indoor decorations and good-luck houseplants popular in Egypt and all over the world in public spaces like malls, offices, and schools^2^. Its importance lies in the easily grown just in water with minimum requirements of care. Low light tolerance attracts positive energy with the beauty of its coordination in vases. It contributes to overcoming air and environmental pollution^3^.

However, lucky bamboo is affected by several fungal diseases, where the root rot, basal stem rot, and wilt disease complex are considered the main constraint for its industry^3,4^. Initial symptoms included a few yellowing rotted areas on the roots, a visible brown discoloration, and rotted areas on the base of the stems as the cortical tissues showed a distinct brown discoloration of water-soaked spots. These lesions became longer up and down and had-dark brown centers with a brown margin, and eventually severe wilting resulted in a significant loss of lucky bamboo plants^5,6^.

*Rhizoctonia solani* is one of the most destructive fungal pathogens that affect large numbers of ornamental crops causing root rot, basal stem rot, and wilt worldwide^7–9^. It can attack many ornamental plants, particularly during production and/or post-harvest storage latently, where it can lie dormant for many years^7,10,11^. Subsequently, fungicides such as Moncut, Rizolex-T, and Topsin-M are used to control the diseases^12–14^. Furthermore, increasing fungicide inputs has several negative consequences, *i.e.,* the development of pathogen resistance to the fungicides, and the killing of the beneficial microorganisms^15^, besides the negative effects of environmental pollution^16,17^.

*R. solani* growth can be controlled by the application of biological controls (bioagents) including the use of microorganisms or their antibiotics offers an attractive alternative for the use of fungicides or management of plant diseases without the negative impact of chemical control. Such bioagents competitively can be colonized plant parts and stimulate the growth and/or reduce the incidence of the plant disease^17,18^. The bioagents *Trichoderma viride*, *T. harzianum*, *Aspergillus niger*, *Penicillium* spp., and *Bacillus subtilis* were observed in in vitro conditions against the tobacco sore shin pathogen, *R. solani* the percentage inhibition values of 70, 67, 57, 50 and 44%, respectively^19^. *T. harzianum* culture filtrates reduced root-rot incidence and damping-off caused by *R. solani* in eggplants, followed by *T. viride*, *P. fluorescens* and *B. subtilis*^20^. Some strains of *P. fluorescens* and *B. subtilis* observed a maximum inhibition of *R. solani* in terms of mycelial growth and sclerotial germination^21^. The bioagent *T. harzianum* reduced the natural incidence of wilt and wet root rot of chickpea in a field plot, as well as increased the seed yield^22^. In dual culture studies, the highest growth inhibition of *R.solani* that causes sheath blight disease of rice was recorded by *T. harzianum* followed by *T. viride*, *G. virens* and *Trichothecium* sp., while disease severity was highest when crop treated with *Trichothecium* sp*.*, *G. roseum, G. virens,* and *T. viride*^23^.

It was reported that the Bio-Arc and Bio-Zeid as active bioagents are classified as biocides^12,15^. Many studies evaluated the activity of *Trichoderma* spp. and *Bacillus* spp. in reducing the spread of *R. solani* and causing completely inhibited growth^24^. *Trichoderma* spp. and *Bacillus* spp. are antagonistic microorganisms used as the most effective pathogen antagonists among biological control agents for many soil-borne and foliar diseases^25–27^. Furthermore, they have been shown to have an antagonistic effect on a wide range of diseases and used to control *R. solani*^17^. The bioagents based on *Bacillus* spp. including *B. circulans* majorly prevent the growth of plant fungal pathogenic microorganisms including *R. solani*^28^. *Bacillus siamensis* as a biocontrol agent showed antagonistic activity against *Aspergillus niger*^29^.

Therefore, the objectives of the present study were to identify and characterize, morphologically and at the molecular level, the pathogen of root rot, basal stem rot, and wilt disease complex that cause damage and kill lucky bamboo plants, isolate and molecular identification of fungal and bacterial bioagents associated with healthy lucky bamboo, and estimate the potential and efficacy of some biocontrol agents to control the main pathogen, *R. solani*, in vitro, and in vivo under laboratory conditions compared to certain fungicides and biocides.

## Materials and methods

This study has complied with relevant institutional, national, and international guidelines and legislation. This study does not contain any studies with human participants or animals performed by any of the authors. This study was conducted with the permission of the Plant Pathology Research Institute, Ornamental, Medicinal, and Aromatic Plant Diseases Research Department, El-Sabihia Agricultural Plant Protection Research Station, Alexandria, and Faculty of Agriculture, Saba Basha, Alexandria University” during the 2019–2021 period.

### Survey for root rot, basal stem rot, and wilt disease complex on lucky bamboo

During the period of March–May 2019, 226 samples of naturally infected lucky bamboo showing characteristic symptoms of severe root rot, basal stem rot, and wilt disease complex were randomly collected from 630 lucky bamboo plants surveyed at various locations at retail stores, shops, and nurseries in Alexandria, El-Behera, and Giza governorates, Egypt. In all the mentioned sites, the percentages of disease infection were recorded and calculated^30,31^ using the ratings shown in Table 1.

**Table 1:** The rate values for the percentages of disease infection.

Rate value	Percentage of disease infection	Symptoms
0	Healthy plants	Either no spots at the base of the stem, and/or no root discoloration, and/or no leaf yellowed or leaf wilted
1	Slight infection of less than 25%	The diseased spots at the base of the stem account for less than 25% of the entire stem circumference and root discolorations
2	Moderate infections of more than 25%	The diseased spots at the base of the stem account for more than 25% of the entire stem circumference and root discoloration and/or one leaf yellowed
3	Severe infections for more than 50%	The diseased spots at the base of the stem account for more than 50% of the entire stem circumference, and root discoloration and/or vascular discoloration, add 2 ± 1 leaf yellowed and/or 1 ± 1 leaf wilted
4	Very severe infections covering more than 75%	The diseased spots at the base of the stem account for more than 75% of the entire stem circumference and root discolorations and/or more than one leaf wilted or completely dead plants

The percentages of disease infection and disease severity were calculated by the following equations:

$${\text{Disease infection } (}\%) {\text{ = }}\frac{{{\text{No. of infected bamboo plants}}}}{{{\text{Total No. of bamboo (healthy and infected) of units assessed}}}} \times 100$$

$${\text{Disease severity } (}\%) {\text{ = }}\frac{{\sum {{\text{(Total No . of bamboo under scale}} \times {\text{scale degree)}}}}}{{{\text{Scale degrees (4)}} \times {\text{Total No. of bamboo (infected) of units assessed}}}} \times 100$$

### Isolation and identification of the associated fungal species

Seventy-five lucky bamboo plant samples showing symptoms of root rot, stem basal rot and wilt disease complex were collected in the survey, labeled separately in paper bags, and transferred to the laboratory to isolate the associated fungal species on the day following collection. Each sample was thoroughly washed in running tap water and cut into small pieces (4 mm), with half of the tissue being healthy and half being diseased tissue. The surface of the pieces was sterilized by soaking in sodium hypochlorite (5%) for 3 min after being rinsed with sterile distilled water (DW).

The pieces were washed thrice with sterile DW, then dried between two layers of sterilized filter papers, and subsequently placed in Petri dishes with potato dextrose agar (PDA) medium supplemented with 250 µL/mL of streptomycin (Egypt Masters Co. (EMC), Dakahlia, Egypt). The plates were then incubated in darkness at 26 ± 1 °C for 7 days^32^, and the developed fungal colonies were purified by a single spore or hyphal tip technique. The developed fungal species were identified based on morphological and microscopic characteristics^33–35^. The frequency of each fungal isolate was calculated according to the following equation.

$${\text{Fungal frequency } (}\%) {\text{ = }}\frac{\text{No. of isolates of each fungus}}{\text{Total No. of all isolates}}\times {1}00$$

### Pathogenicity tests and molecular identification of the isolated fungal species

#### Pathogenicity tests

Ostensibly healthy-looking lucky bamboo plants, with uniform stem lengths averaging 70 cm, were purchased from a famous private commercial nursery in Cairo. In the laboratory, plants were well-washed several times with running tap water, surface disinfected in 1% sodium hypochlorite for 2 min, then washed several times with running tap water and rinsed with sterilized DW. To ensure that the plants are healthy and free of any pathogens, they were sown in glassware containing 300 ml of sterile DW for 60 days under the laboratory conditions (12 h photoperiod at 26 ± 2 °C with an average relative humidity of 65–70%) before conducting any experiments on them. Furthermore, to reduce bacteria entry from the surrounding air and prevent water evaporation, all glassware is wrapped with a sterilized cotton stopper around the bamboo stem. Before sown, each glassware was cleaned and sterilized in a hot air oven for 2 h at 180 °C and then left to cool^3,36^.

Thirty healthy bamboo plants were placed in 1-L sterile glassware (1 plant/glassware) by dipping 5 cm of basal stems with 300 ml of sterile DW. Fifteen plants were inoculated separately by adding directly an excerpt of *Rhizoctonia solani* mycelial agar plug (0.5 cm diameter) cut from a 7-day-old culture disc at 26 ± 2 °C of the active margins of the fungal culture recovered in the survey. The culture was inserted into a cut in the basal stem segment with a sterile Cork borer. A similar plug of sterile PDA served as the negative control of the remaining 15 bamboo plants. The inoculated areas were then covered with Parafilm strips and to provide wet conditions, the plants were covered with polyethylene plastic bags for 24 h. All bamboo plants were kept for 4 weeks under laboratory conditions. At the end of the test, the percentages of infection and severity of disease were calculated as described above^30,37^.

To ensure that the pathogen was associated with the symptoms, it was re-isolated from the symptoms in artificially infected plant tissues. Subsequently, the developed fungal cultures were purified as described above, then they were identified based on morphological and microscopic characteristics and molecular identification.

#### Molecular identification of the recovered *Rhizoctonia solani* isolates

The most dominant fungal species, i.e.,* R. solani*, was further identified and molecularly characterized by polymerase chain reaction (PCR) amplification and 18S sequencing. The cultures were sent to the “Molecular Biology Research Unit, Assiut University for DNA extraction using a Patho Gene-spin DNA/RNA extraction Kit provided by Intron Biotechnology Company, Korea. Samples of fungal DNA were then sent to SolGent Company, Daejeon, South Korea for PCR and 18S sequencing. PCR was performed using ITS1 (forward) and ITS4 (reverse) primers, which were incorporated into the reaction mixture^38^. Primers have areas with universal primer pairs including ITS1 (5'-TCC GTA GGT GAA CCT GCG G-3'), and ITS4 (5'-TCC TCC GCT TAT TGA TAT GC-3'). The purified PCR products (amplicons) were sequenced using the same primers, but with ddNTPs added to the reaction mixture^39^. Identification of isolate was confirmed by the obtained sequences of the amplified regions and analyzed using “the Basic Local Alignment Search Tool (BLAST) from the National Center of Biotechnology Information (NCBI) website (http://www.ncbi.nlm.nih.gov). Molecular Evolutionary Genetics Analysis version 5.05 of MegAlign (DNA Star) software was used to perform the alignments. The identified phylogenetic tree based on ITS sequences of rDNA of the isolated fungal strain aligned with closely related sequences was accessed from the GenBank.

### Control studies

#### Isolation of the associated biocontrol agents of the healthy lucky bamboo

Isolation of the antagonistic micro-organisms on healthy lucky bamboo plants was conducted after the incubation period of bamboo plants, which exceeded 60 days under the laboratory conditions, to ensure that they are healthy plants free of any apparent infestations^3^. Plant parts of 2 cm long were cut from the basal stem with roots of 20 bamboo plants, then 1 g of each sample was taken and processed as mentioned above for the isolation and microscopic and molecular identification of the fungi.

The isolation of the associated bacteria was conducted according to Abdel–Rahman^40^, with minor modifications. The crushed plant parts were sterilized with sodium hypochlorite solution (10%) for 2 min, immersed in sterilized DW for 2 min, and washed thoroughly several times with sterilized DW. Afterward, 1 g from each sample tissue (parts of each basal stem and root) was mixed with 9.9 ml of sterile saline (sterile physiological water from NaCl, 9 g/l) individually and squashed into the sterilized mortar and pestle to homogenize. Then, the solution was diluted serially in the sterile saline individually for each sample up to 10^6^ CFU/ml^41^. A loopful (1 ml) of the resulting suspension of each dilution was spread on nutrient agar (9 ml of NA) plates and incubated for 24 h at 30 ± 1 °C. In order to obtain new separate colonies, single colonies were selected and purified on fresh NA plates on the base of variance in morphology, e.g., color, size, and shape. After 24–48 h at the same temperature, pure bacterial colonies appeared, then identified according to their morphological and biochemical characteristics^42,43^, and performed using standard methods by Agricultural Laboratories Company (Agro Lab, Sadat City, Egypt).

#### Molecular identification of the associated biocontrol agents of the healthy lucky bamboo

DNA was extracted from the isolation of pure cultures of fungi and bacteria ABT DNA mini extraction Kit (Applied Biotechnology Co. Ltd, Egypt) for molecular characterization of the Internal Transcript Spacer (ITS) region using Polymerase Chain Reaction (PCR) amplification, "2X Red master Mix (Applied Biotechnology Co., Egypt), and Oligonucleotide (Alpha DNA Co, Canada)"^44^. The ITS DNA region of these isolates was amplified via PCR using universal primers. The following is the optimized thermal profile for PCR: Initial denaturation (95 °C for 3 min), Denaturation (95 °C for 30 s., Annealing (50 °C for 30 s.), Extension (72 °C for 90 s.), and Final extension (72 °C for 5 min.), Repeating for 35 cycles.

To confirm the targeted PCR amplification, five μl of the PCR product was electrophoresed along with 100 bp DNA molecular weight 1% agarose gel containing ethidium bromide (0.5 μg/ml) at constant 80 V for 30 min in 1X TAE buffer. The amplified product was visualized as a single compact band of expected size under UV light and documented by the Samsung Note 4 smartphone.

Sequencing of the PCR product for the amplified PCR products was submitted to Solgent Co Ltd (South Korea) for gel purification and sequencing. The resulted sequences were trimmed and assembled in Geneious software (Biomatters). Consequently, the trimmed sequences were identified by search in the basic local alignment search tool (BLAST) in GenBank.

Furthermore, phylogenetic analysis by nucleotide sequences was downloaded from GenBank and aligned with the identified sequences, using MAFFT alignment^45^. Phylogenetic trees were constructed using the neighbor-joining method, employing the Tamura–Nei Model^46^. The trees were assessed using 1000 bootstrap replicates.

#### Evaluation of the isolated bioagents against *Rhizoctonia solani *in vitro

The in vitro inhibition effects of the recovered bioagent isolates, i.e., the fungal isolate *Clonostachys rosea,* and the three recovered bacterial isolates *Bacillus circulans*, *B. siamensis,* and *Ochrobactrum anthropi*, were tested against the highly aggressive isolate of *R. solani* AUMC15120, which recovered in the conducted survey. This was done in comparison with the untreated inoculated control as a negative control as well as three fungicides and two biocides as positive controls (Table 2).

**Table 2:** Fungal and bacterial bioagents, fungicides, and biocides, used in the present study against *Rhizoctonia solani*, with their trade name, formulation, common and chemical name, recommended dose, and their sources.

T	Isolate code and/or trade name (formulation)	Types	Fungal and bacterial name and/or common name [IUPAC chemical name]˜ and/or active ingredients	Rate and/or recommended dose	Source or manufacturer
Control untreated		*Rhizoctonia solani* AUMC15120			
T1	TF-2	Fungus	*Clonostachys rosea*	1 × 10^7^/ml**	Isolates
T2	Seq6	Bacterium	*Bacillus circulans*	1 × 10^7^*CFU/ml^$^	Isolates
T3	Seq1	Bacterium	*B. siamensis*	1 × 10^7^ CFU/ml	Isolates
T4	Seq2	Bacterium	*Ochrobactrum anthropi*	1 × 10^8#^ CFU/ml	Isolates
T5	Moncut (25% wp)	Fungicide	Flutolanil 25% [α,α,α-trifluoro-3′-isopropoxy-o-toluanilide]	2.0 g/l	Nihon Nohyako, Japan
T6	Rizolex-T 50%	Fungicide	Tolclofos-(ethyluro-o-dimethyl) [O-2,6-dichloro-p-tolyl O, O-dimethyl phosphorothioate]	1.5 g/l	Sumitomo Chemical, Cairo
T7	Topsin-M (70% wp)	Fungicide	Thiophanate-methyl [dimethyl 4,4′-(o-phenylene) bis(3-thioallophanate)]	1.0 g/l	Cerexagri, Hokko, Nippon Soda, Cairo
T8	Bio-Zeid (2.5% wp)	Biocide	*Trichoderma album* 25 × 10^6^ spores/g	2.5 g/l	Organic Biotechnology, Cairo
T9	Bio-Arc (6% wp)	Biocide	*Bacillus megaterium* 25 × 10^6^ cell/g	2.5 g/l	

T = treatments, **conidia spores/ml^47^, *^48^**,** ^$^ = serial dilutions cell (CFU/ml) namely colony-forming units ml^−1^, ^#^^49^, ˜IUPAC = International Union for Pure and Applied Chemistry.

The tested fungal and bacterial bioagents as well as the tested fungal and bacterial biocides were grown under laboratory conditions. For biocides, a disc of filter paper 5 mm in diameter, impregnated with Bio-Zeid (*Trichoderma album* 25 × 10^6^ spores/g) suspension, was inoculated at the recommended rate into the middle of a Petri dish, then incubated at 26 ± 2 °C for 7-days, while; bacterial isolates and/or Bio-Arc were grown individually in 250 ml flasks that each contained 50 ml of the NA medium. Then after the incubation for 72 h, they were used for the streaked method.

#### Antagonistic potential of the isolated bioagents against *Rhizoctonia solani*

Isolated bioagents were screened for their antagonistic potential against *R. solani* by double culture assay using solid PDA plates^50^. Each PDA plate was divided into two equal halves, and at a distance of 1 cm from the edge of every plate from opposite sides, plates were inoculated on one side with a mycelial disc (5 mm diameter) taken from the margins of the active growing *R. solani* of 7-day-old PDA cultures. Furthermore, on the other opposite side of each plate at 1 cm distance from the plate edge, a 5 mm of the tested fungal bioagent (*C. rosea*) or fungal biocide plug (taken from advanced margins of 7-day-old PDA cultures) was placed.

Likewise, a filter paper disc of 5 mm diameter, impregnated with each fungicide separately was placed^51^. Additionally, the bacterial bioagent isolates and/or Bio-Arc were treated by a streaking method^3^. Untreated check (control) plates were treated without bioagents and/or fungicides.

Five replicate plates were used for each treatment and incubated at 26 ± 2 °C until the untreated control mycelium (*R. solani*) totally colonized the plate. At that time, radial growth in plates for each treatment was measured to determine the percentage of the growth inhibition by calculating the percentage of radial growth reduction in diameter mycelia of *R. solani*^3,50^ as follows^3,44^:

$${\text{Growth inhibition } (}\%) {\text{ = }}\frac{{{\text{GD}}^{{\text{u}}} - {\text{GD}}^{{\text{T}}} }}{{{\text{GD}}^{{\text{u}}} }} \times 100$$

In which (GD^u^) is the radial growth diameter of pathogen mycelia untreated (*R. solani*) in the control plate, apart from the bioagents (cm), and (GD^T^) is the radial growth diameter of the treated pathogen mycelia (*R. solani*) toward the bioagents and/or fungicides (cm).

### The in vivo evaluation of the efficacy of the isolated bioagents to control *Rhizoctonia solani* on lucky bamboo in vase under laboratory conditions

The antagonistic effect of fungal isolate (*C. rosea*), bacterial isolates (*B. circulans*, *B. siamensis*, and *O. anthropi*), fungicides and biocides (Table 2) that showed maximum inhibitory activity in vitro against the highly aggressive *R. solani* AUMC15120 isolate. i.e., *Clonostachys rosea* AUMC15121″, *Bacillus circulans* MW441316, *B. siamensis* MW441318, *Ochrobactrum anthropi* MW441316, Moncut, Bio-Zeid and Bio-Arc were tested in vivo on lucky bamboo in the vase under laboratory conditions at the recommended dose (Table 2).

Inocula of the fungal bioagent *C. rosea* were prepared as follows; flasks of 100 ml of potato dextrose liquid (PDL) were inoculated by adding directly a sporulating mycelial agar plug (0.5 cm diameter) taken from a 7-day-old culture active margins of *C. rosea* and incubated at 22 ± 2 °C with photoperiods 12 h under white fluorescent lamps until sufficient mycelial growth was obtained. Subsequently, the collected mycelial growth was blended with 100 ml of sterile distilled water for 1 min. The conidia spore’s concentration was adjusted using the hemocytometer technique^47^, viz 10^7^ conidia spores/ml inoculum concentrations.

The bacterial suspensions of bioagents and biocides were cultured on Luria–Bertani (LB) broth medium at flasks containing 100 ml of medium for 2 days at 30 ± 1 °C and shaken at 200 rpm to harvest bacteria. Then the bacterial concentrations in the solution were adjusted by serial dilution with sterile saline individually 1 × 10^7^ CFU/ml^48^, and 1 × 10^8^ CFU/ml^49^.

All treatment were conducted one day after inoculation with *R. solani* (as mentioned above in the pathogenicity testing) to 120 glassware vessels planted with healthy lucky bamboo separately. Sterile distilled water was used instead of the treatments as an untreated control. All treatments and control were kept for 4 weeks under laboratory conditions. At the end of the trial, the percentages of disease infection and disease severity (%) were recorded as mentioned above. Subsequently, the percentage decrease or increase from the untreated control was calculated^52^. In addition, the percentage of treatment efficiency was evaluated using the formulas proposed^53^, respectively:

$${\text{Decrease or increase }(}\%) {\text{=}}\frac{\text{Disease infection (\%) of control} - \text{Disease infection (\%) of treatment}}{\mathrm{Disease\,\, infection\,\, (\%)\,\, of\,\, control}}\times 100$$

$${\text{Treatment efficiency } (}\%) {\text{=}}\frac{\text{Disease severity (\%) in untreated control}- \text{Disease severity (\%) in treatment}}{\mathrm{Disease\,\, severity\,\, (\%)\,\, in\,\, untreated \,\,control}}\times 100$$

### Statistical analysis

The obtained data of the tested treatments (bioagents, fungicides, and biocides) against the growth of *R. solani* were statistically analyzed using Statistix program, by using the software analysis of variance with one-way ANOVA test^54^ and compared with the untreated control. All trials were carried out with a randomized complete block design (RCBD). Each treatment with five replicates, each replicate contains 3 plates and/or 3 glassware. Each glassware contains one bamboo plant. Comparisons among the means were evaluated using the least significance difference (LSD) at a 5% level of probability.

## Results

### Survey for root rot, basal stem rot and wilt disease complex on lucky bamboo

**Figure 1:**
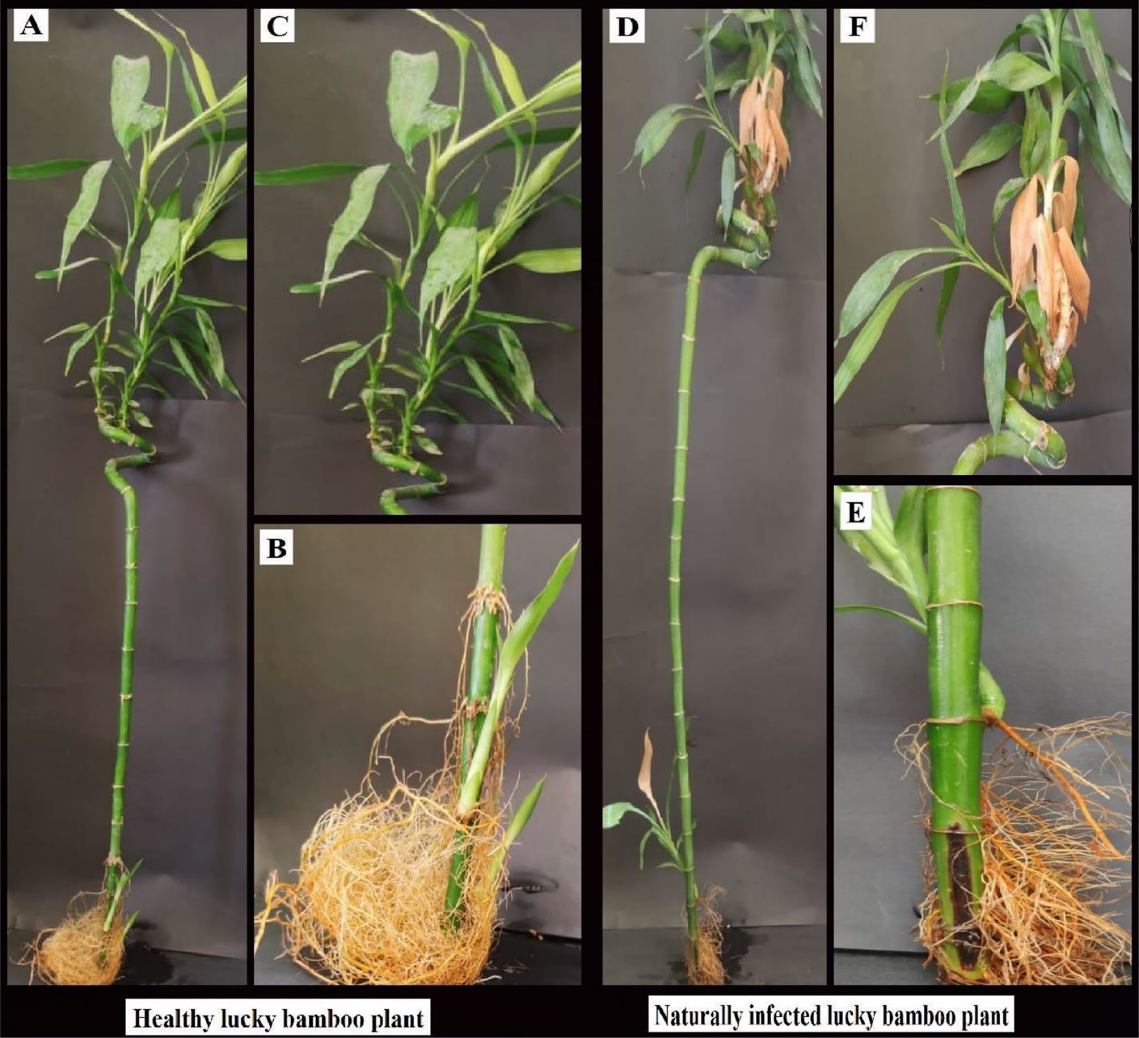
Appearance healthy and naturally infected for lucky bamboo (*Dracaena sanderiana* hort. ex. Mast.), and Symptoms of basal stems and roots (**A** & **D**) complete plant form (**B**) healthy basal stem and -brown lesions) (**F**) few yellowing roots, (**C**) healthy leaves, (**E**) basal stem rot, root rot (infected basal stem have dry of brown to reddish leaves).

During the period March–May 2019, the conducted survey for root rot, basal stem rot and wilt disease complex on lucky bamboo plants, typical symptoms were identified (Fig. 1) in the surveyed retail stores, shops, and nurseries from different locations in Egypt (Alexandria, El-Behera, and Giza governorates). However, the surveyed locations and governorates showed considerable variations in the percentage of infection and disease severity % (Table 3).

**Table 3:** Percentage of naturally disease infection and disease severity of root rot, basal stem rot, and wilt disease complex of lucky bamboo plants surveyed in different locations in the three Egyptian governorates.

Governorates	Total no. of surveyed plants	No. of infected plants	Disease infection (%)	Disease severity (%)
Alexandria	220	103	47.67^A^* ± 9.25	34.86^a^ ± 5.22
Behera	210	056	26.98^B^ ± 4.50	35.19^a^ ± 6.10
Giza	200	067	33.14^B^ ± 8.49	31.53^a^ ± 6.54
Means	210	075	35.93	33.86
L.S.D. at 0.05	–	–	11.571	7.261

*Values within the same column followed by a different letter (s) are significantly different at 0.05 level of probability.

From the data in Table 3, the mean percentage of disease infection was the highest in plants collected from Alexandria (47.67%) followed by Giza (33.14%) and El-Behera (26.98%) Governorates. However, the disease severity for the three surveyed governorates ranged between 35.19and 31.53%, with no significant differences between the surveyed governorates (Table 3).

### Fungi associated with root rot, basal stem rot, and wilt disease complex of lucky bamboo plants

**Figure 2:**
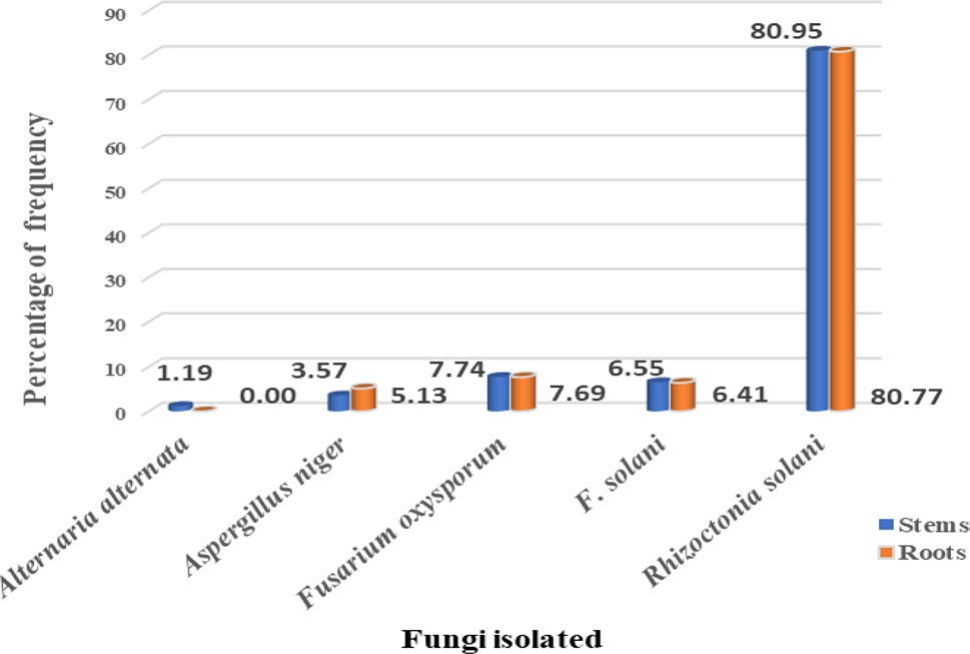
Frequency of fungi isolated from diseased root rot and basal stem rot of lucky bamboo located in different locations in three Egyptian governorates.

Data in Table 4 show that the five fungal isolates *R. solani*, *F. oxysporum*, *F. solani, Aspergillus niger*, and *Alternaria alternata* were found to be associated with the surveyed lucky bamboo samples with symptoms of root rot, basal stem rot, and wilt disease complex. However, *R. solani* isolates were the most frequent among fungal species recovered and constituted 80.89% of the total isolates while the other fungal species showed frequencies lower than 10% (Fig. 2).

**Table 4:** The overall mean of frequent fungal species recovers isolated from diseased root rot and basal stem rot of lucky bamboo after beingsurveyed from different locations in the three Egyptian governorates.

The isolated fungi	No. of isolates		Overall mean	
	Stems	Roots	Total	Frequency (%)
*Alternaria alternata*	002	00	002	00.81
*Aspergillus niger*	006	04	000	04.07
*Fusarium oxysporum*	013	06	019	07.72
*F. solani*	011	05	016	06.50
*Rhizoctonia solani*	136	63	199	80.89
Total counts of fungi	168	78	246	100

### Pathogenicity test

**Figure 3:**
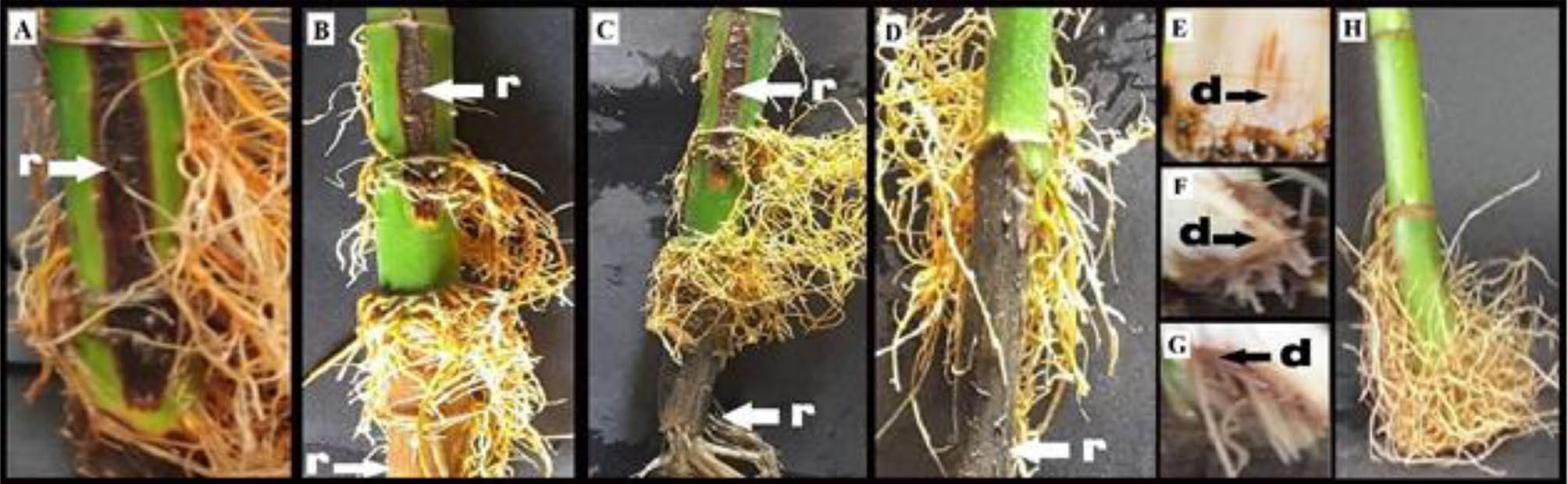
Symptoms of basal stem rot, root rot at natural and artificial infestation caused by *Rhizoctonia solani* during pathogenicity test of lucky bamboo (*D. sanderiana* hort. ex. Mast.) in vitro, (**A**) naturally infected, and (**B**, **C** & **D**) symptoms of dieback of the rotting basal stem party after artificial infected (r) rotting brown discoloration at the middle of the basal stem, (**E**, **F**, and **G**) longitudinal sections of the basal stem, (d) internal brown discoloration of the basal stem's, and (**H**) uninfected control.

It is evident in Table 5 that the tested fungal species recovered in the survey were able to incite root rot and basal stem rot to different degrees. However, data in Table 5 showed that *R. solani* isolates were the most pathogenic occurred on lucky bamboo and exhibited a 100% percentage of infection with 76.67% disease severity. Meanwhile, *F. oxysporum* and *F. solani* showed percentages of infection of 33.33% and 26.67% with 35.0% and 27.5% disease severity, respectively. The other two fungal species *Aspergillus niger*, and *Alternaria alternata* showed an infection percentage of 20% or less with a disease severity of 15% or less (Table 5). Moreover, Fig. 3 illustrated the symptoms of basal stem rot and root rot when artificial infestation by *R. solani* during the pathogenicity test and compared it with that of natural infestation of lucky bamboo plants.

**Table 5:** Pathogenicity of fungal species on lucky bamboo plants in vase under laboratory conditions, after 4 weeks survey.

The isolated fungi	Disease infection (%)	Disease severity (%)
Uninfected control	00.00 C	00.00 c
*Alternaria alternata*	13.33 bc*	15.00 bc
*Aspergillus niger*	20.00 bc	12.50 bc
*Fusarium oxysporum*	33.33 b	35.00 b
*F. solani*	26.67 bc	27.50 bc
*Rhizoctonia solani*	100.00 a	76.67 a
LSD at 5%	27.929	26.088

*Values are means of five replicates; each replicate contains three bamboos (plant/vase). values within the same column followed by a different letter(s) are significantly different at 0.05 of probability.

### Molecular characterization of the recovered *Rhizoctonia solani* isolates

**Figure 4:**
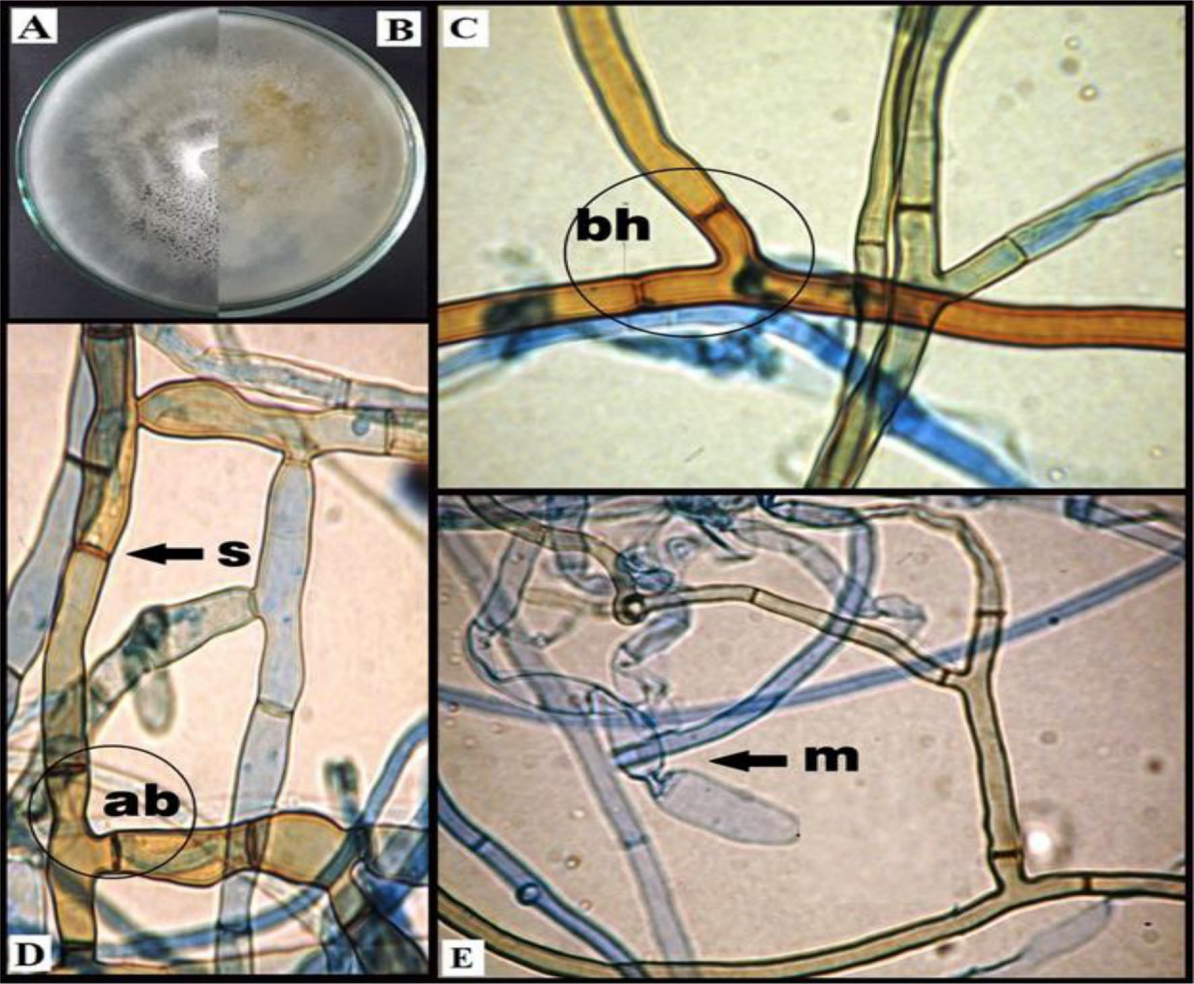
Morphological and microscopic characteristics of *Rhizoctonia solani* fungus isolated from basal stems and roots of bamboo plants, (**A**, **B**): mycelial of *R. solani* on PDA medium after 10 days (A: top surface and A: lower surface) for the petri dish, and (**C**, **D** & **E**) microscopic traits of *R. solani* of the hyphal stage at a typical angle branching (ab) with numerous branching filaments of specialized hyphae composed of compact cells called monilioid cells (m), branching hyphae (bh) and septum (s).

**Figure 5:**
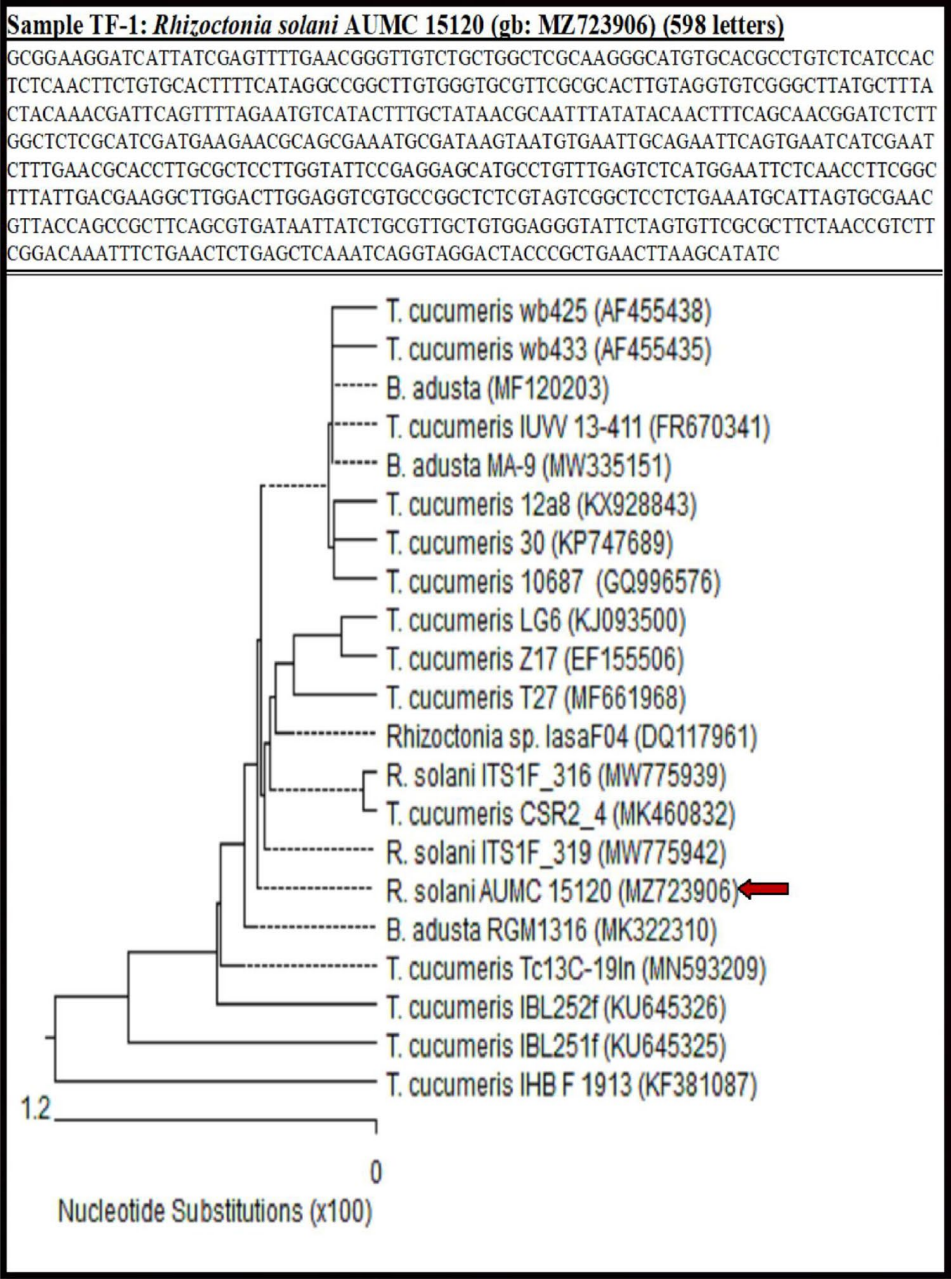
Phylogenetic tree based on ITS sequences of rDNA of the fungal strain isolated in the present study (*Rhizoctonia solani* AUMC 15,120) aligned with closely related sequences accessed from the GenBank, (R. = *Rhizoctonia*, T. = *Thanatephorus* and B. = *Bjerkandera*), (https://www.ncbi.nlm.nih.gov/nuccore/gb:MZ723906).

After the morphological and microscopic characteristics of *R. solani* isolates (Fig. 4), the molecular identification of *R. solani* isolates was conducted by PCR amplification and 18S sequencing. The tested strain showed 100% identity and 100% coverage with several strains of *R. solani* accessed from the GenBank (Fig. 5). The fungus was putatively identified as *Rhizoctonia solani* AUMC 15120 (GenBank accession No. MZ723906). Furthermore, the phylogenetic tree identified showed that *R. solani* (AUMC 15120) aligned with closely related sequences accessed from the GenBank, i.e.,* Thanatephorus cucumeris* which is the teleomorph (sexual stage) of *R. solani* (Fig. 5).

### Control studies

#### Isolation and identification of the associated biocontrol agents of the healthy lucky bamboo

**Figure 6:**
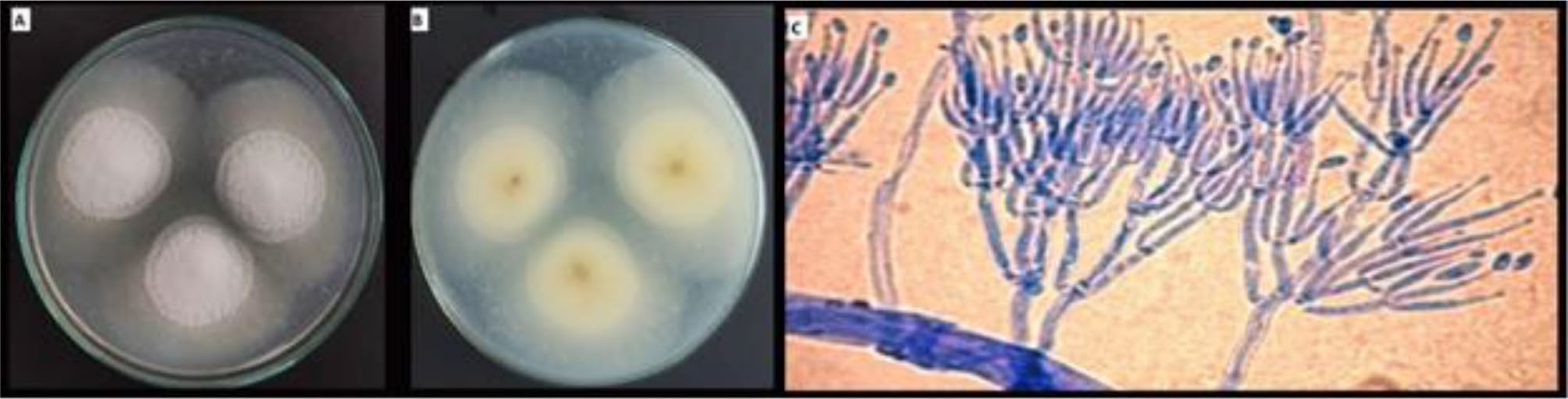
Cultural and morphological characteristics of the bioagent, *Clonostachys rosea*; isolate AUMC15121 of healthy lucky bamboo (*D. sanderiana*) plants: Colony on PDA after seven days, (**A**) top surface, (**B**) lower surface, and (**C**) conidiophores and Conidia.

**Figure 7:**
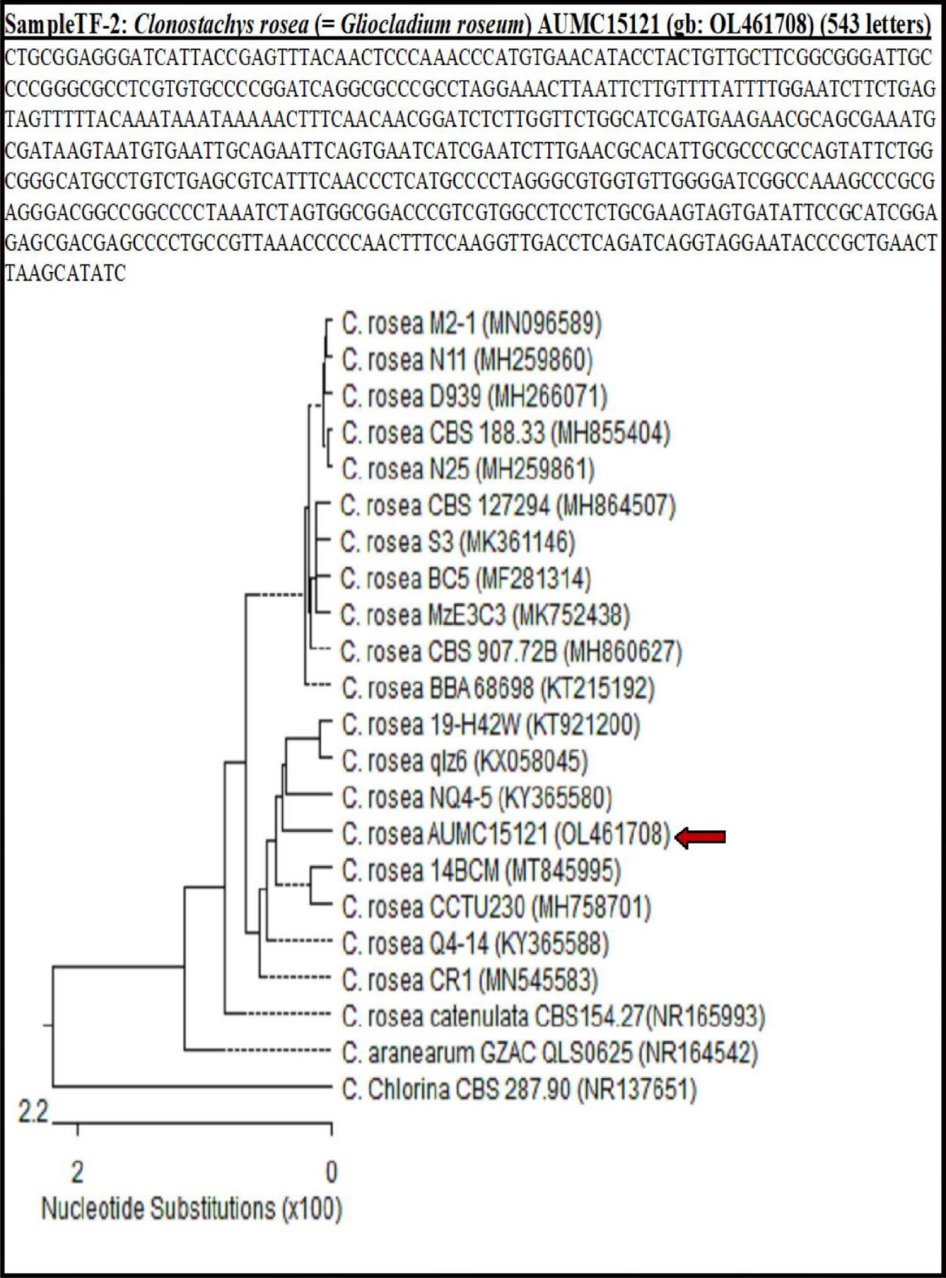
Phylogenetic tree based on ITS sequences of rDNA of the fungal strain isolated in the present study (*Clonostachys rosea* AUMC15121–OL461708) aligned with closely related sequences accessed from the GenBank (C. = *Clonostachys*).

**Figure 8:**
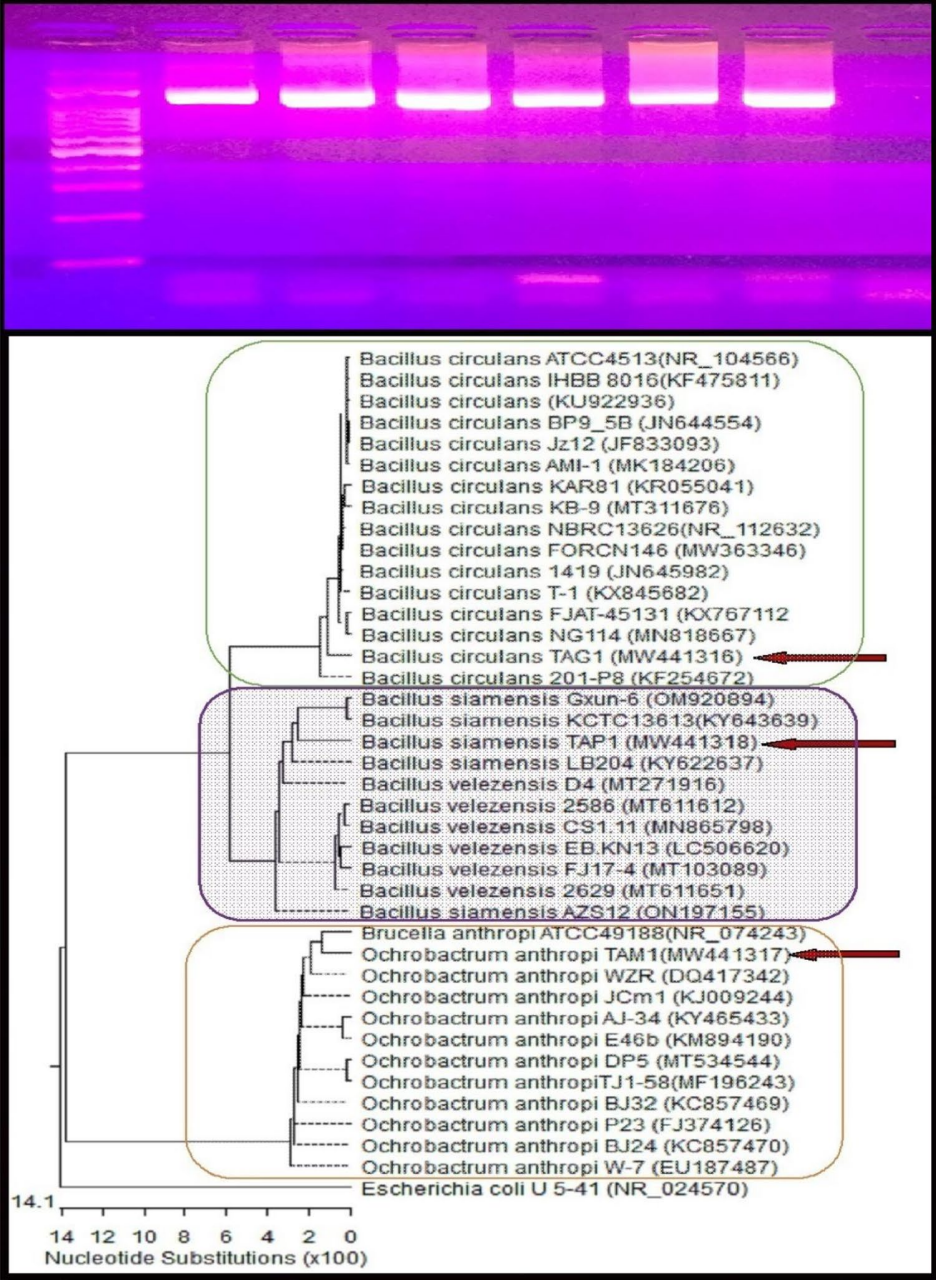
The amplified product as a single compact band of expected size under UV light, and phylogenetic tree based on 16S sequences of rDNA of the bacterial strains isolated in the present study (*Bacillus circulans* TAG1, *Bacillus siamensis* TAP1, and *Ochrobactrum anthropi* TAM1, arrowed) aligned with closely related sequences accessed from the GenBank. *Escherichia coli* is included in the tree as an outgroup strain.

Four biological control agents were isolated from healthy lucky bamboo plants (Table 6). Three of them were bacterial strains (*Bacillus circulans*, *B. siamensis*, and *Ochrobactrum anthropi*), and one fungal strain (*Clonostachys rosea*)*.* The isolated fungus was morphologically investigated (Fig. 6) and all bioagents were characterized at the molecular level (Figs. 7 and 8).

**Table 6:** Code and GenBank accession number for fungal and bacterial bioagent isolates recovered from healthy lucky bamboo plants.

Code	Fungal and Bacterial isolates	GenBank Acc. No	Partial sequence
TF-2	*Clonostachys rosea*	AUMC15121˜	strain OL461708 5.8S ribosomal RNA gene
Seq6	*Bacillus circulans*	MW441316*	strain TAG1 16S ribosomal RNA gene
Seq1	*B. siamensis*	MW441318**	strain TAP1 16S ribosomal RNA gene
Seq2	*Ochrobactrum anthropi*	MW441317***	strain TAM1 16S ribosomal RNA gene

˜https://www.ncbi.nlm.nih.gov/nuccore/OL461708, *https://www.ncbi.nlm.nih.gov/nuccore/MW441316, **https://www.ncbi.nlm.nih.gov/nuccore/MW441318, ***https://www.ncbi.nlm.nih.gov/nuccore/MW441317.

The molecular characterization showed that *Clonostachys rosea* AUMC 15,121 fungal isolate exhibited 99.63–99.63% identity and 100% coverage with several strains of *C. rosea* (Fig. 7). The three bacterial isolates (*B. circulans*, *B. siamensis*, and *O. anthropi*) amplified products were visualized as single compact bands of expected size under UV light (Fig. 8). *B. circulans* TAG1 showed 97.60–97.69% identity and 99% coverage with several strains of the same species recorded in the GenBank including the type material *B. circulans* strain NBRC 13626 while *B. siamensis* TAP1 showed 95.68–95.97% identity and 97% coverage with different strains of *B. siamensis* recorded in the GenBank.

However, *O. anthropi* TAM1 exhibited 96.92–97.11% identity and 99% coverage with other strains accessed from the GenBank encompassing the type strain *Brucella anthropic* ATCC49188 which is registered in the GenBank as the type strain of *O. anthropi*, Family Brucellaceae (Fig. 8).

#### The in vitro evaluation of the isolated bioagents against *Rhizoctonia solani*

The isolated bioagents from healthy lucky bamboo were evaluated for their antagonistic potential against *R. solani* recovered from lucky bamboo, in vitro and in vivo and compared with some other fungicides and biocides.

**Figure 9:**
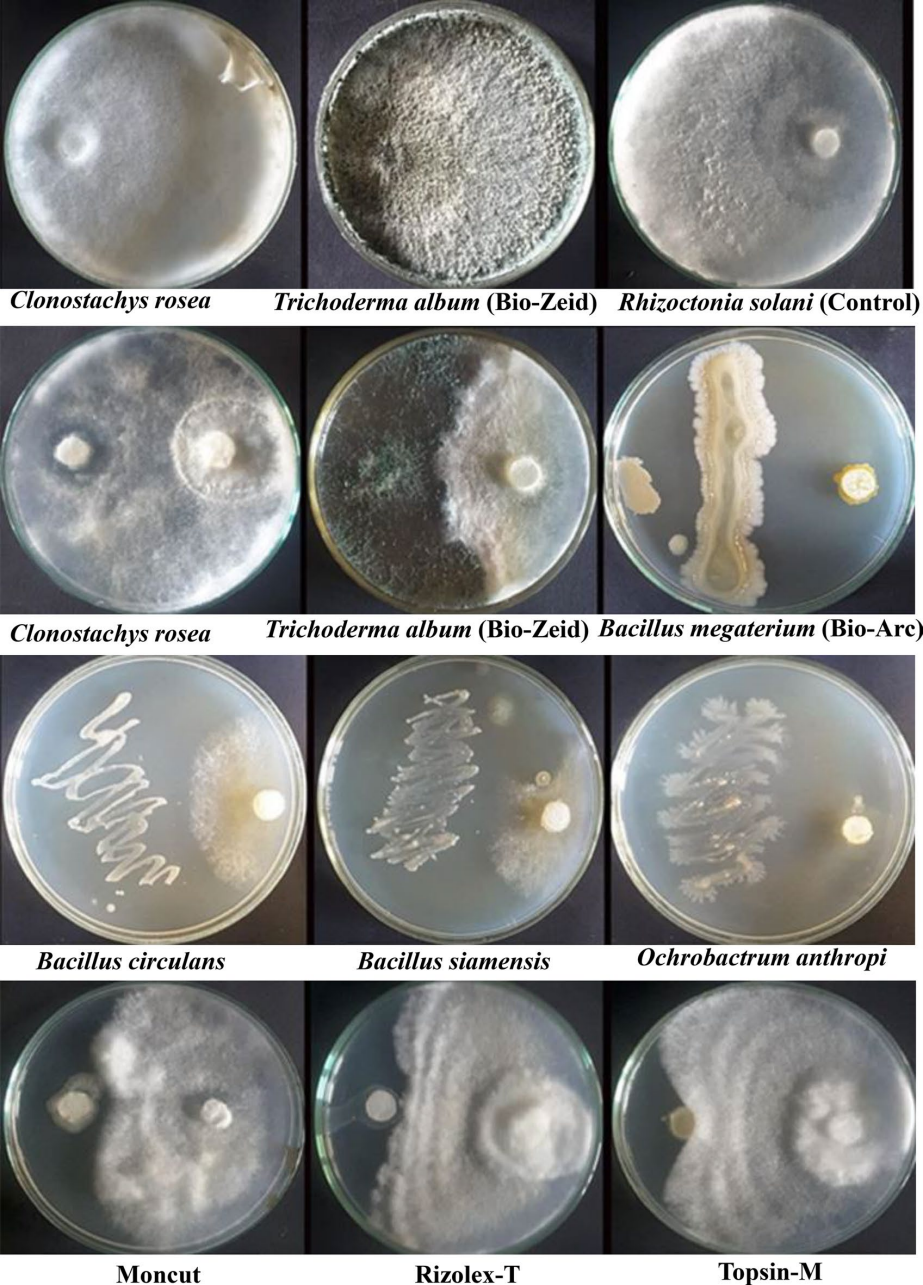
Direct confrontation dual culture plate assays for determining the antagonistic potential of fungus and bacterial isolates, fungicide, and biocide against *Rhizoctonia solani* AUMC 15120 by inhibition of pathogen mycelial growth compared to the untreated control plates in vitro. The cultures were incubated at 26 ± 2 °C.

It is evident in Fig. 9 that all tested bioagents and the tested fungicides and biocides affected the tested *R. solani* isolate to different degrees. However, data in Table 7 showed that the highest *R. solani* colony growth inhibition was presented by bioagent *O. anthropi* isolate (85.11%), followed by biocide Bio-Arc (83.78%), while bioagent *C. rosea* showed 65.33%. The bioagents *B. siamensis* and *B. circulans* exhibited 64.44, and 60.44%, respectively, while the biocide Bio-Zeid showed an inhibition value of 43.11%. However, the least growth inhibition was recorded by Rizolex-T with 34.22% and Topsin-M with 28.67% of inhibition (Table 7).

**Table 7:** The in vitro inhibition of the tested bioagents, and certain fungicides and biocides on mycelia growth (cm) of *Rhizoctonia solani* grown on PDA after six days at 26 °C.

Treatments	Mycelia growth (cm) ± SD	Inhibition (%) ± SD
Control (*Rhizoctonia solani*)	9.00^A^ ± 0.00	0.00^g^ ± 0.00
*Clonostachys rosea*	3.12^F^* ± 0.29	65.33^b^ ± 3.18
*Bacillus circulans*	3.56^E^ ± 0.21	60.44^c^ ± 2.30
*B. siamensis*	3.20^F^ ± 0.10	64.44^b^ ± 1.11
*Ochrobactrum anthropi*	1.34^G^ ± 0.18	85.11^a^ ± 2.02
Moncut (2 g/l)	5.34^D^ ± 0.18	40.67^d^ ± 2.02
Rizolex-T (2 g/l)	5.92^C^ ± 0.24	34.22^e^ ± 2.65
Topsin-M (1 g/l)	6.48^B^ ± 0.13	28.67^f^ ± 1.45
Bio-Zeid (*Trichoderma album* at 2.5 g/l)	5.12^D^ ± 0.25	43.11^d^ ± 2.77
Bio-Arc (*Bacillus megaterium* at 2.5 g/l)	1.46^G^ ± 0.27	83.78^a^ ± 3.00
LSD at 5%	0.1316	1.4705

*Values are means of five replicate plates, values within the same column followed by different letter(s) are significantly different at 0.05 of probability.

#### The in vivo evaluation of the efficacy of the isolated bioagents to control *Rhizoctonia solani* on lucky bamboo in vase under laboratory conditions

**Figure 10:**
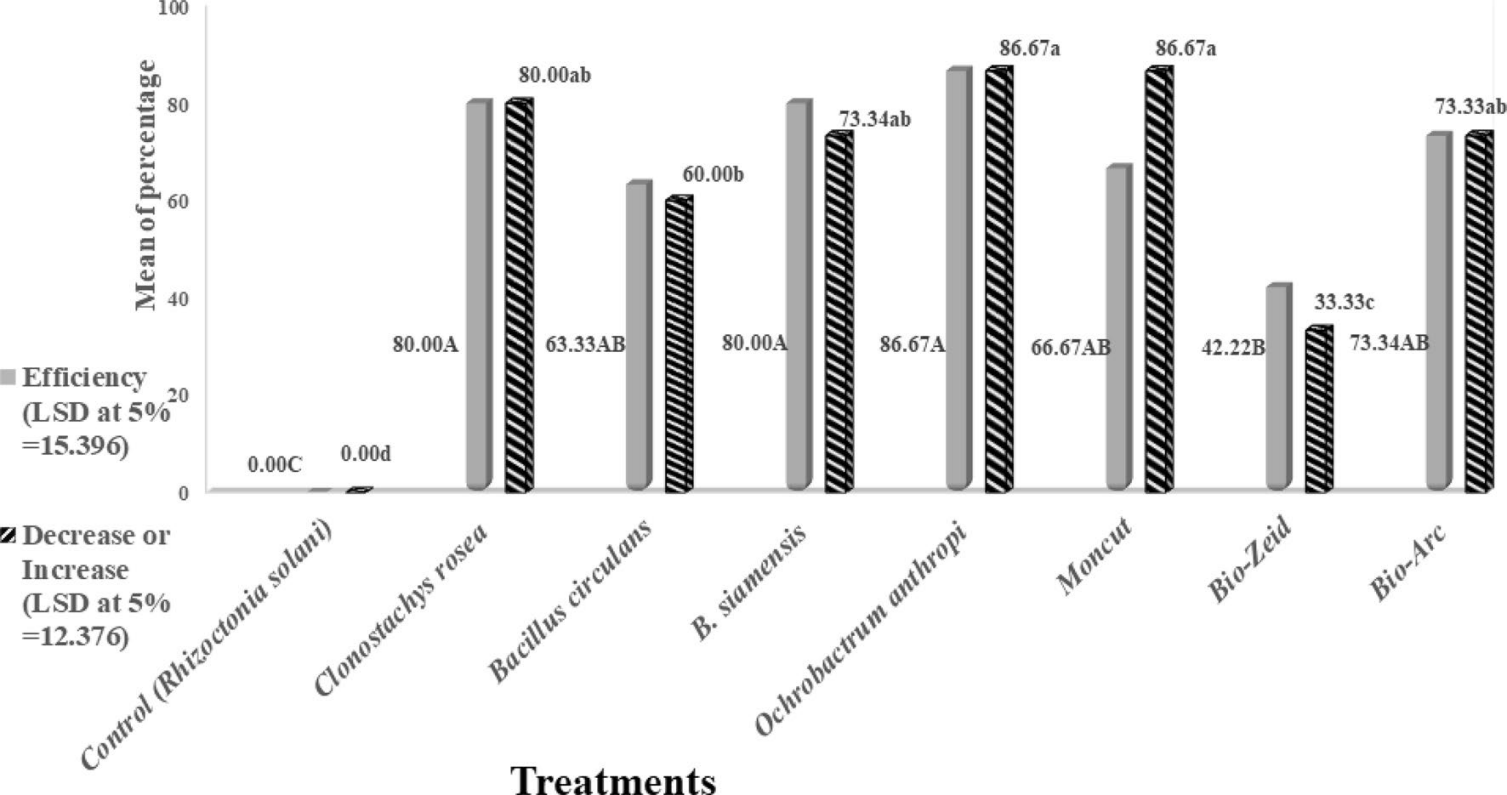
In vivo effect of treatments on the mean percentage of decrease or increase in infection with *Rhizoctonia solani* and the average percentage of their effectiveness against the same fungus compared to the untreated control of the lucky bamboo in a vase under laboratory conditions.

The most effective treatments revealed in vitro were further tested in vivo in vases under laboratory conditions. Data in Table 8 showed the disease incidence and disease severity caused by *R. solani*. A decrease or increase in infection with and treatment effectiveness against *R. solani* on bamboo artificially inoculated under laboratory conditions for 4 weeks after inoculation and treatments are shown in Fig. 10.

**Table 8:** In vivo effect of the tested bioagents and certain fungicides and biocides on disease incidence and severity caused by artificial infection with *Rhizoctonia solani* on lucky bamboo in vase, after 4 weeks under laboratory conditions.

Treatments	Disease incidence %	Disease severity %
Control (*Rhizoctonia solani*)	100.00^A^* ± 00.0	75.00^a^ ± 08.3
*Clonostachys rosea* (1 × 10^7^/ml)	20.00^D^ ± 08.2	15.00^c^ ± 12.3
*Bacillus circulans* (1 × 10^7^ CFU/ml)	40.00^C^ ± 17.8	27.00^bc^ ± 13.6
*B. siamensis* (1 × 10^7^ CFU/ml)	26.67^CD^ ± 17.8	15.00^c^ ± 11.6
*Ochrobactrum anthropi* (1 × 10^8^ CFU/ml)	13.33^D^ ± 08.2	10.00^c^ ± 09.0
Moncut (2 g/l)	13.33^D^ ± 08.2	21.00^bc^ ± 08.3
Bio-Zeid (*Trichoderma album* at 2.5 g/l)	66.67^B^ ± 17.8	43.33^b^ ± 16.7
Bio-Arc (*Bacillus megaterium* at 2.5 g/l)	26.67^CD^ ± 18.2	20.00^bc^ ± 13.6
LSD at 5%	15.351	11.547

*Values are means of five replicates, each replicate contains three bamboos (plant/vase) of each treatment; Values followed by different letter(s) within the same column are significantly different at 0.05% level of probability.

All the tested treatments (Table 8) significantly decreased the infection percentage and disease severity compared to the inoculated untreated control. However, the best effect was obtained by *O. anthropi* and the fungicide Moncut, with the lowest disease incidence value of 13.33%, followed by *C. rosea* (20%) compared to the control (100%). The lowest disease severity (10%) was obtained by *O. anthropi* followed by *Clonostachys rosea* (15%) and *B. siamensis* (15%) compared to the control (75%). However, *B. circulans* showed 40% and 27% while, Bio-Zeid (*Trichoderma album*) showed the least effect with values of 66.67% and 43.33%, for both disease parameters, respectively (Table 8).

Furthermore, all the tested treatments significantly decreased the disease incidence and gave positive increasing responses regarding the treatment efficiency in comparison to the untreated control (Fig. 10). *O. anthropi* afforded the highest disease reduction to other isolates, and it gave the highest efficiency at 86.67%. Bio-Zeid (*T. album*) yielded the lowest reduction of the pathogen and the lowest mean percentage of efficiency (viz 33.33 and 42.22%, respectively, both disease parameters) compared to the infected untreated control.

## Discussion

Lucky bamboo (*D. sanderiana* hort. ex. Mast.) is well known in Egypt and other parts of the world as an important indoor ornamental plant. Meanwhile, it possesses importance viz the ease of care, low light tolerance, attract positive energy with its beauty and contribution to overcoming air and environmental pollution, etc.^2,3,55,56^. However, several reports indicated that lucky bamboo plants are seriously affected by root rot, basal stem rot and wilt disease complex^3,8,57,58^. Furthermore, several works indicated the presence of latent diseases in imported bamboo plants^5,6,58^.

The present study confirmed these reports in a survey conducted during the period of March–May 2019 in nurseries, warehouses, and shops in different locations(Alexandria, El-Behera, and Giza), in Egypt. The percentage of disease infection was the highest in Alexandria (47.67%) followed by Giza and El-Behera Governorates with 33.14% and 26.98%, respectively. In addition, the disease severity for the three surveyed governorates ranged between 35.19% (El-Behera Governorate) and 31.53% (Giza Governorate). These findings are not unexpected as disease infection and severity were of the highest values in such governorates with high humidity, Alexandria, and El-Behera Governorates. These results are in harmony with other investigators^2,3,8^.

In the present work, five fungal species were found to be associated with the surveyed lucky bamboo samples showing root rot, basal stem rot, and wilt disease complex. These fungal species were *R. solani*, *Fusarium oxysporum*, *F. solani, Aspergillus niger*, and *Alternaria alternata*. Most of these fungi were isolated from lucky bamboo by several researchers^3–6^. However, *R. solani* isolates were the most dominant among fungal species recovered with a percentage of 80.89%. These findings also are in harmony with other investigators^10,11^. *R. solani* is one of the most dangerous and highly destructive fungal pathogens affecting many ornamental crops and several treatments and trials using natural products and bioagents have been done to control its growth^7,8,59–64^.

Adoption of biological control is one of the crucial approaches currently at the forefront and is strongly desired for sustainable agriculture^18^. Several features of using biocontrol agents have been reported as one eco-friendly alternative or a supplemental way of reducing the use of toxic fungicides in ornamental plants^65^. Therefore, it is important to come up with new biological control products for eco-friendly and sustainable efficient benefits^17^. Furthermore, increasing levels of plant resistance using biological inducers isolated from the same plant is a new sustainable strategy for plant disease control. Identification of biocompatible isolates for managing lucky bamboo root rot, basal stem rot, and wilt caused by *R. solani* would be a beneficial contribution to disease management with low toxicity and minimal potential risk to the environment.

The present study supported this phenomenon, where four bioagents *Clonostachys rosea*, *Bacillus circulans*, *B. siamensis*, and *Ochrobactrum anthropi* were isolated from healthy lucky bamboo plants and were identified at the molecular level and significantly showed potential to inhibit *R. solani *in vitro as well as in vivo on lucky bamboo plants in vase treatments compared with the untreated inoculated control (negative control) as well as certain fungicides and biocides. All the tested bioagents (*C. rosea*, *B. circulans*, *B. siamensis*, and *O. anthropi*) as well as the tested fungicides and biocides, i.e., Moncut (2 g/l), Rizolex-T (2.0 g/l), Topsin-M (1 g/l), Bio-Zeid (2.5 g/l), and Bio-Arc (2.5 g/l), significantly inhibited the in vitro growth (colony diameter) of the tested *R. solani* isolate to different degrees.

Meanwhile, the in vivo experiment supported the in vitro results for the most effective treatments. All the tested treatments significantly decreased the percentage of infection and disease severity, showed significantly decreased disease incidence, and gave positive increasing responses regarding the treatment efficiency compared to the inoculated untreated control. The bioagent *O. anthropi* showed the highest effect, i.e., the lowest disease incidence and disease severity compared to the untreated inoculated control. This was not significantly different from the fungicide Moncut, and the bioagent *C. rosea*. These results are consistence with other investigators^14,15,28,66–69^.

The bioagent fungus *Clonostachys rosea* (*Gliocladium roseum*) is proven to be a promising and strong biocontrol agent for a variety of plant pathogens including fungal, nematodes, and insects. It has been recorded as an aggressive parasite against many fungi of excellent bioagent in plant diseases through techniques like nutrient competition and hyperparasitism^47,70,71^. Also, mechanisms of *C. rosea* biocontrol are represented in the production of a wide range of volatile organic compounds which are toxic to organism's pathogens, and it could be due to the induction of plant defense responses systems, e.g., excretes cell wall‐degrading enzymes, antifungal secondary metabolite production, or leading to induced systemic resistance (ISR)^72,73^.

*O. anthropi*, a siderophore-producing bacteria, is used as a potential biological control agent. It has shown a pretty antagonistic activity against many fungi such *Botrytis cinerea*, *Colletotrichum orbiculare*, and *Fusarium oxysporum,* etc.^66,68,74^.

Meanwhile, it has been indicated that *Bacillus circulans* emergence of chitinase enzyme activity against various plant pathogenic fungi, and also the *B.* spp. as a biocontrol has many benefits, including viz increasing mineral uptake, nitrogen fixation, and growing a strong and disease-resistant plant^28,41,75,76^. Additionally, *B. siamensis* succeeded as a biocontrol agent by producing antifungal compounds^29^ such as poly-γ-glutamic acid^67^, and it was proven to enhance plant growth and heighten plant growth-promoting qualities^27^.

The most important findings of this research are the isolation and identification of the pathogenic fungus *R. solani* from the symptoms of natural infection of bamboo plants. Furthermore, isolation and identification of four associated biocontrol agents of the healthy bamboo plants were done based on morphological and microscopic characteristics and molecular phylogenetic analysis and GenBank accession. To the best of our knowledge, this is the first report of the isolation of each *R. solani* AUMC 15120, *Clonostachys rosea* AUMC15121, OL461708 5.8S, *Bacillus circulans* TAG1, 16S MW441316, *B. siamensis* TAP1, 16S MW441318, and *Ochrobactrum anthropi* TAM1, 16S MW441317 of lucky bamboo (*Dracaena sanderiana* hort. ex. Mast.) in Egypt.

The present study supported the identification of biocompatible isolates for managing lucky bamboo root rot, and basal stem rot caused by *R. solani* that would be a beneficial contribution to disease management with low toxicity and minimal potential risk to the environment.

## Conclusions

This is the first report of the isolation and identification of the pathogenic fungus *Rhizoctonia solani* from the symptoms of natural infection of bamboo, and, four associated biocontrol agents of the healthy lucky bamboo. They were identified based on morphological and microscopic characteristics, molecular phylogenetic analysis and GenBank accession. Consequently, the bioagents *Ochrobactrum anthropi* MW441317 at 1 × 10^8^ CFU/ml, or *Clonostachys rosea* AUMC15121 at 1 × 10^7^/ml proved to be efficient to control the growth of *R. solani* which causes root rot, and basal stem rot on lucky bamboo plants, which are as effective as the most efficient Moncut fungicide. This work recommends the use of biocides isolated from the same plant to combat *R. solani*, despite the efficiency of some fungicides in reducing infection rates and severity for sustainable protection of the environment.

## Data Availability

All data generated or analyzed during this study are included in this published article. The datasets analyzed during the current study are available from the corresponding author on reasonable request.
